# Fusion of Neuro-Signals and Dynamic Signatures for Person Authentication

**DOI:** 10.3390/s19214641

**Published:** 2019-10-28

**Authors:** Pradeep Kumar, Rajkumar Saini, Barjinder Kaur, Partha Pratim Roy, Erik Scheme

**Affiliations:** 1 Institute of Biomedical Engineering, University of New Brunswick, Fredericton, NB E3B 5A3, Canadaescheme@unb.ca (E.S.); 2 Department of Computer Science & Engineering, Indian Institute of Technology, Roorkee 247667, India; rajkumarsaini.rs@gmail.com (R.S.); proy.fcs@iitr.ac.in (P.P.R.)

**Keywords:** electroencephalography (EEG), authentication, biometrics, dynamic signature, identification, smartphone

## Abstract

Many biometric systems based on physiological traits such as ones facial characteristics, iris, and fingerprint have been developed for authentication purposes. Such security systems, however, commonly suffer from impersonation attacks such as obfuscation, abrasion, latent samples, and covert attack. More conventional behavioral methods, such as passwords and signatures, suffer from similar issues and can easily be spoofed. With growing levels of private data readily available across the internet, a more robust authentication system is needed for use in emerging technologies and mobile applications. In this paper, we present a novel multimodal biometric user authentication framework by combining the behavioral dynamic signature with the the physiological electroencephalograph (EEG) to restrict unauthorized access. EEG signals of 33 genuine users were collected while signing on their mobile phones. The recorded sequences were modeled using a bidirectional long short-term memory neural network (BLSTM-NN) based sequential classifier to accomplish person identification and verification. An accuracy of 98.78% was obtained for identification using decision fusion of dynamic signatures and EEG signals. The robustness of the framework was also tested against 1650 impersonation attempts made by 25 forged users by imitating the dynamic signatures of genuine users. Verification performance was measured using detection error tradeoff (DET) curves and half total error rate (HTER) security matrices using true positive rate (TPR) and false acceptance rate (FAR), resulting in 3.75% FAR and 1.87% HTER with 100% TPR for forgery attempts.

## 1. Introduction

Privacy of data has been a concern since ancient times. Camouflaging information with a practice called steganography was used to send data from one place to another, hiding information in the hair of a messenger, within a wax table, or using secret vanishing ink. Since these early strategies, many more methods have evolved to secure data, including digital watermarking, the use of puzzles, patchworks, chameleon schemes, etc. With the emergence of the internet, there has been a revolution in access to information over remote broadband or wireless networks, re-emphasizing the need for authentication mechanisms to secure access to information [1].

The evolution of network and mobile technologies has also facilitated numerous ways of accessing information on-the-go, including the now ubiquitous mobile phone. Various traditional authentication systems (knowledge or token based), such as passwords, are commonly used but it is becoming difficult for many users to remember numerous combinations of complex alpha-numeric characters. Problems are also faced when ID cards or PINs get lost [2]. Several biometrics have been developed such as fingerprint, palm, eye, face, and voice, but these too can suffer from known threats like covert attacks, obfuscation, abrasion, and latent samples [3].

To counter these privacy attacks, researchers have proposed different kinds of biometric verification systems using neurological signals [4,5]. These signals are generated by electrical spikes that occur as millions of neurons in the brain respond to internal or external stimuli. Various non-invasive techniques such as electroencephalograph (EEG), functional magnetic resonance imaging (fMRI), and magnetoencephalography (MEG) have been used to analyze these brain signals. It has been reported that a person’s neurosignals, in response to a given task, are unique and relatively consistent [6]. By utilizing combinations of neurons from different parts of the brain, a robust biometric authentication system may therefore be possible. Indeed, in 2014, Rocca et al. [7] proposed an identification system using EEG signals. They fused the spectral coherence between different brain regions and achieved maximum accuracy while fusing frontal lobe regions in eyes open and eyes closed scenarios.

Conversely, dynamic signatures are also commonly used to implement person authentication systems for financial, banking, and retail market applications [8]. Such behavioral property-based authentication systems, which evaluate not only the signature, but how it was created, are widely accepted by society because they are not easily lost or forgotten. Zareen et al. [9] proposed a mobile based signature verification system by collecting signatures of 25 people using a smart phone; the x,y coordinates and pressure information were used as features for the verification process using a back-propagation neural network.

Today’s smartphones are equipped with many applications, including financial transactions, healthcare, and remote authentication, that until recently would have been considered too secure for use on mobile devices. Many of these applications therefore require digital signature or PIN passwords for user authentication. However, these security mechanisms are not robust to spoofing attacks that aim to gain unauthorized access to places or information. Moreover, many other technologies and locations require even more stringent access control. A secure mechanism that could combine both behavioral and physiological information to improve the robustness of access to these systems is therefore warranted.

In this paper, we present a novel framework to implement a robust person authentication system using a combination of dynamic signatures and EEG signals. The EEG signals are recorded using an EEG-Android API while an individual signs their signature on the screen of a mobile device. The main contributions of the paper are therefore as follows:

1. We present a multi-modal framework to simultaneously capture dynamic-signatures and EEG signals for the development of a mobile user authentication system.
2. We demonstrate an implementation of identification and verification tasks using both unimodal and multimodal approaches with a bi-directional long short-term memory neural network (BLSTM-NN) classifier.

## 2. Background

Presentation attacks, or imposter attacks, occur when a forged version of a signature with near-genuine characteristics is presented to the system to impersonate someone’s identity [10]. Given the importance of detecting such attempts, presentation attack detection (PAD) methodologies have been the focus of recent research on dynamic signature based authentication systems. Raul et al. [11] analyzed the robustness of dynamic signature biometrics against presentation attacks and proposed two different metrics, the number of strokes and signing time, to counter them. Presentation attacks can be analyzed in various ways depending on the amount of information and training available to the forger. In [12], the authors performed a PAD analysis of dynamic hand signature datasets. It was found that not only the type of information, but also the training and effort in performing the forged signature, had a large impact on the system’s performance. For example, a skilled forgery detection scheme for dynamic signature based versification systems was developed using kinematic theory and sigma-log normal features [13]. However, their scheme required skilled forgeries to train the skilled detector. Likewise, the authors in [14] proposed a forgery detection system for static signatures using fuzzy modeling. Forgery detection was based on angle features extracted using a grid method which were fuzzified by an exponential membership function.

In recent years, the effectiveness of multimodal systems has been noted because of their advantages in providing greater security in comparison to unimodal systems [15]. In such systems, the forger must mimic more than one biometric trait concurrently, providing added difficulty and a level of redundancy in the system. Such an approach—using feature level fusion for dynamic signature and a fingerprint based multimodal biometric system—was proposed in [16]. Different features such as pressure, speed, and pen up/down movements were extracted from the dynamic signature data and fused with the bifurcation and ridge features of the fingerprint using a sum rule. Similarly, Sujatha et al. [17] used the discrete wavelet transform (DWT) encoding technique to integrate multiple biometric traits such as iris, palm print, face, and signature. A multimodal gait recognition system was proposed in [18], where a decision fusion approach was used to combine the results of inertial sensors (accelerometer, gyroscope, and magnetometer) and video data. The fusion process was carried out using a evolutionary algorithm that yielded gait recognition accuracies of 91.3%. In this work, we combine dynamic signatures with EEG signals to capture the ability of the user to generate the signature both accurately and naturally.

## 3. Methodology

### 3.1. Data Collection

**Figure 1:**
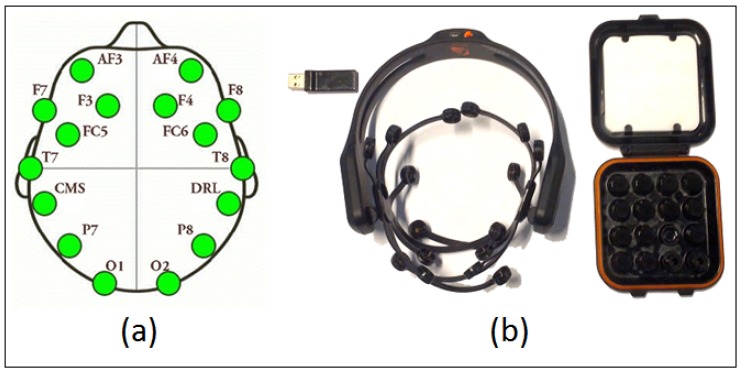
Emotiv EPOC+ device: (**a**) Electrode position over the scalp as per International 10–20 system; (**b**) sensor and the associated accessories.

Fifty-eight healthy volunteers (23 ± 3 years old, 18 female) participated in the experiment. All subjects gave written informed consent in accordance with the Declaration of Helsinki prior to their participation in the experiment. An Emotiv Epoc + neuro-headset with 14 sensors + 2 references was used to capture EEG information while subjects signed their signatures on mobile phone screen. The Emotiv device records EEG data with a sampling frequency of 128 Hz. The EEG signals from the 14 channels (i.e., AF3, AF4, F3, F4, F7, F8, FC5, FC6, P7, P8, T7, T8, O1, and O2) depicted in Figure 1 were recorded on the mobile phone using an EEG-Android API while the live signatures were captured using a custom graphical user interface (GUI) on a Samsung Galaxy Note 9 phone running the Android 8.1 operating system. The physical dimensions of the device are (161.9 × 76.4 × 8.8 mm) with a display size of 160 mm. The input device was an S Pen stylus (5.7 × 4.35 × 106.37 mm) with 12 bit pressure resolution and a tip size of 0.7 mm. The mobile device was placed on a horizontal table with the screen facing upward while the signer sat in a chair wearing the Emotiv EEG device. In order to familiarize themselves with using the system, each signer was asked to perform 2–5, or until they felt comfortable. The signers were also asked to write their full names in signature style to preserve the privacy of their original signatures.

Of the 58 users, 33 were arbitrarily treated as genuine, whereas the remaining 25 were treated as forged users. All users were instructed to remain calm and were asked to keep their body steady while performing the signatures with their eyes open. In a single data collection session, each genuine user recorded 10 signatures with a resting time of 10–15 s between consecutive signatures. Thus, a total of 330 (33×10) samples of dynamic signatures were recorded with simultaneous EEG signals. For imposter verification testing, 25 users attempted to forge the signatures of each genuine user twice, resulting in 1650 (i.e., 25×2×33) forged samples of signatures. Before forging a signature, the forgers were shown images of the genuine signature for 1–2 minutes and asked to practice forging it at least 10 times. Using this approach, the forgers were able to build their knowledge and training before data collection.

**Figure 2:**
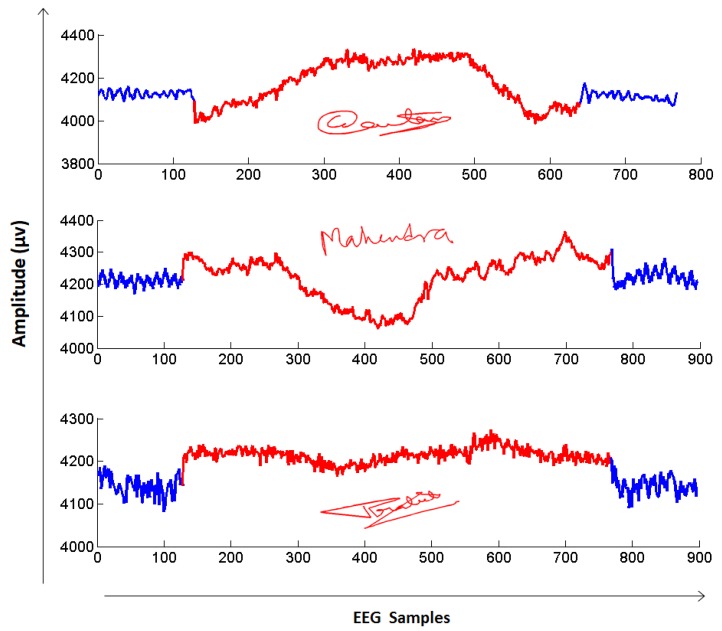
Analysis of the changes in electroencephalography (EEG) while signing for three different users. Blue signals depict the EEG variation before and after the signature whereas red signals correspond to the EEG of users while signing. Note: EEG signals are plotted for just one electrode positioned at ‘AF4’. Data were recorded using an Emotiv Epoc+ device with a sampling frequency of 128 Hz (each sample denotes ~7.8 ms).

Examples of the changes in a single EEG sensor while signing their signature are shown for three different users in Figure 2. A large and distinct variation in EEG can be seen between the users. The blue portions of the signals in Figure 2 depict the EEG preceding and following the act of signing whereas the red signals correspond to the EEG during the period that the users were signing. From these figures, it is clear that the EEG changes in concert with the handwritten strokes of a signature. This simple example serves as motivation for exploring EEG as part of the proposed EEG-signature scheme.

**Figure 3:**
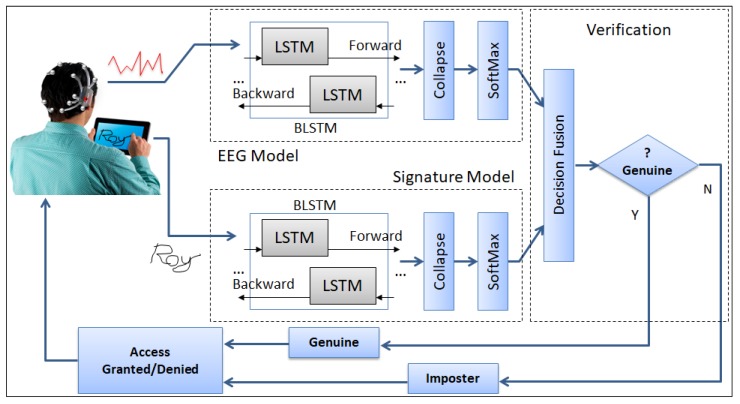
Block-diagram of the proposed authentication framework.

The mobile application captured the time-series of x,y coordinates of the signature as drawn on the mobile screen. A block diagram of the approach is depicted in Figure 3, where the EEG headset is mounted over the head of a person drawing a signature on a mobile phone while EEG signals are recorded simultaneously. Next, the multimodal inputs were modeled using two different BLSTM-NN classifiers. The final output layer was governed by the Softmax function that compresses a *K*-dimensional vector z of arbitrary real values to a *K*-dimensional vector σ(z) of real values in the range [0, 1] that sum to 1. The Softmax outcome was then fused using different decision fusion techniques to build identification and verification models.

### 3.2. Feature Extraction

EEG signals were explained by DFTfeatures whereas the dynamic signatures were described using writing direction-based angular features as follows.

#### DFT Features Extracted from EEG Signals ($F_{D}$)

DFT has been used previously to effectively process EEG signals as it splits the signals into constituent sinusoidal waves of different frequency bands. In this work, a Hanning window, seen in Equation (1), was applied to filter the EEG signals using α=0.5, as discussed in [19]. Next, the DFT coefficients were computed from the filtered data as in Equation (2),

(1) $$W\left(x\right)=\alpha -\left(1-\alpha \right)\;cos\left(\frac{2\pi x}{N}\right)\;\;\;\;for\;\;0\leq x\leq N-1$$

(2) $$F_{u}=\sum_{x=0}^{N-1}f_{x}\;\;e^{-\;j\frac{2\pi }{N}ux}\;\;\;\left(u=0,1..N-1\right)$$

where *f* is a discrete and finite function of *x* of length *N*. The exponent $e^{-\;j\frac{2\pi }{N}ux}$ is given as in Equation (3).

(3) $$e^{-\;j\frac{2\pi }{N}ux}=\cos\left(-\frac{2\pi }{N}ux\right)+j\sin\left(-\frac{2\pi }{N}ux\right)$$

**Figure 4:**
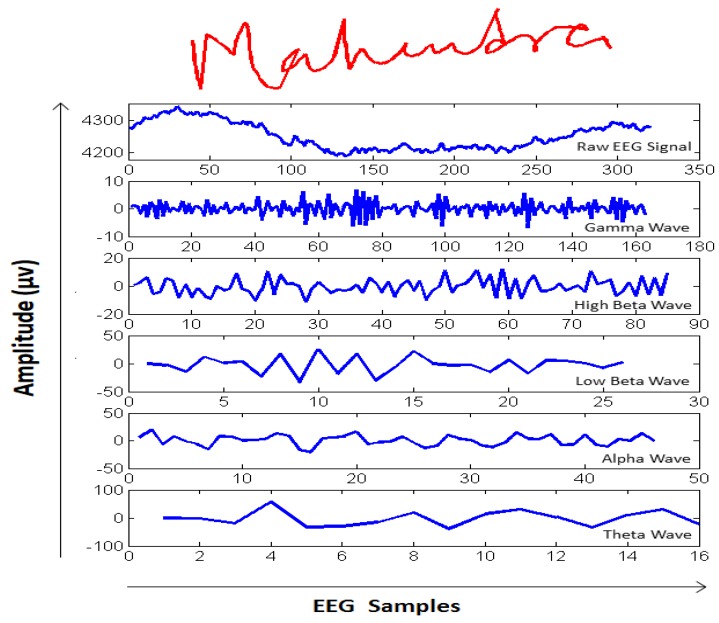
An example of EEG signal decomposition using DFT analysis corresponding to the signature shown above the axes in red. From top to bottom: raw EEG signal, Gamma, High Beta, Low Beta, Alpha, and Theta waves.

DFT splits an input signal into different frequency bands, namely, *Theta (4–8 Hz)*, *Alpha (9–12 Hz)*, *Low Beta (13–16 Hz)*, *High Beta (17–25 Hz)*, and *Gamma (26–40 Hz)*. In particular, gamma band waves are an important feature for developing an EEG-based person authentication system [20,21]. Therefore, in this work, the feature vector ($F_{D}$) was comprised of *Gamma* band waves from all 14 channels. These features were concatenated and fed into the BLSTM neural network for classification. A DFT analysis of a raw EEG signal, along with the user’s signature, is depicted in Figure 4.

### 3.3. Dynamic Signature Features ($F_{S}$)

For dynamic signatures, the feature vector $F_{S}$ consisted of the time-series of the raw signature *T*, and the writing direction of the signature trajectories *W*, detailed below. $F_{S}$ can therefore be represented as $F_{S}$ = {*T*,*W*}.

#### 3.3.1. Signature Trajectory (*T*)

The raw time-series of position data recorded by the mobile device, representing the dynamic signature trajectory, was used as a feature as represented by Equation (4), where ($x_{i},y_{i}$) denotes the coordinate positions corresponding to the *i*th sample of the signature trajectory.

(4) $$T=(x_{i},y_{i})\;where\;i=1,2,..,n$$

#### 3.3.2. Writing Direction (*W*)

**Figure 5:**
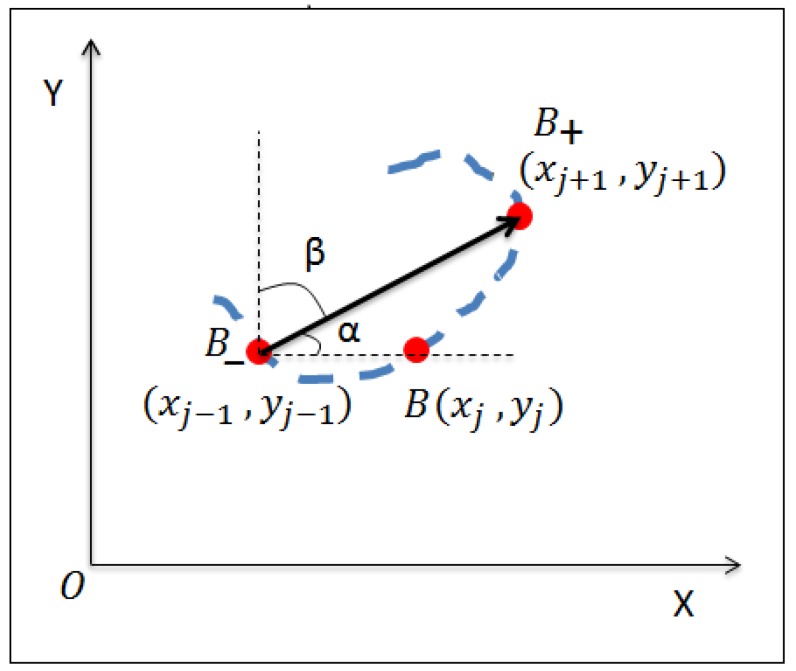
Computation of writing direction for a point $B(x_{j},y_{j})$ on the signature trajectory with the help of neighboring points.

Writing direction based features have been widely used in dynamic signature based identification and verification systems [22,23]. The writing direction of a point $B(x_{j},y_{j})$ was calculated as the slope of the line joining the two neighboring points, namely, $B_{-}\left(x_{j-1},y_{j-1}\right)$ and $B_{+}\left(x_{j+1},y_{j+1}\right)$. The vector $\vec{B}$ forms angles α, β with the coordinate axes, as depicted in Figure 5, which are used as the features for *W*.

### 3.4. Person Identification and Verification Using BLSTM-NN

BLSTM-NN has been widely used to model temporal data in handwriting [24] and gesture recognition [25] problems. BLSTM-NNs have also been reported to outperform hidden markov model (HMM) classifiers in speech and handwriting recognition problems because HMM assumes the probability of each observation in the current state, causing difficulty when modeling contextual information. However, BLSTM-NN has the ability to process the input sequence in both directions, forward and backward, using two hidden layers [26]. The classifier has one output layer connected to both hidden layers, thus getting access to bi-directional information for every point in the input sequence.

In this work, a BLSTM-NN classifier was used for sequence classification of digitally acquired signatures and EEG signals for person identification and verification. The network was trained separately for feature vectors $F_{S}$ and $F_{D}$ using a cross entropy error based objective function. The output layer was implemented using a Softmax function which ensures that the normalized output of the network was between 0 and 1, and summed to 1 for each time step, as is a standard for 1 of *C* classification [26]. The network has *C* output units, one for each class of the signature and EEG sequence. The cross entropy error (E) for *C* classes can be computed as in Equation (5).

(5) $$E\;=\;-\sum_{(x,z)\epsilon S}\;\sum_{c=1}^{C}z_{c}\ln r_{c}$$

where *x* and *z* denote the input sequence and the target sequence, respectively. (x,z) represents the input pair from the training set *S*, and *r* denotes the probability scores such that the input belongs to a particular class.

Both the signature and EEG-based BLSTM-NN classifiers were trained with initial weights selected from a random distribution in the range of [−0.1,0.1]. A momentum of 0.9 was selected with a learning-rate of 1e−4. The selection of training order was chosen randomly at the start of each training epoch and the weights were updated after each signature sequence.

#### 3.4.1. Multimodal Decision Fusion Approach

Decision fusion approaches have been used to successfully combine the results of multiple uni-modular schemes without requiring the synchronization of features. Consequently, independent systems can be separately modeled and trained before being fused. In this work, *Sum Rule, Borda Count, and Max Rule* classifier combination techniques were used [27]. While the different methods vary slightly, they all combine the decisions of the different constituent classifiers using the rank or confidences of the class outputs. The details are as follows.

##### Sum Rule

The sum rule assumes statistical independence between multiple representations and that posterior probabilities computed by the individual classifiers do not deviate from the prior probabilities [28]. The rule assigns a class ‘*c*’ to the test pattern from the pool of ‘*m*’ (1,2,3...m) different classes using Equation (6), where $\vec{x_{i}}$ denotes the feature vector presented to the $i^{th}$ classifier (1,2,3..R). The term $P(w_{j}|\vec{x_{i}})$ denotes the posterior probability of the input belonging to class $w_{j}$ given feature vector $\vec{x_{i}}$.

(6) $$c=\arg\;\max_{j}\sum_{i=1}^{R}P\left(w_{j}|\vec{x_{i}}\right)$$

##### Borda Count

Borda count is a consensus-based voting system wherein the candidate with the maximum number of votes is ranked highest (say *n*, where *n* is the total number of candidates). The remaining candidates are ranked based on their rank, with second highest being assigned n−1, and so on. This per voter ranking is summed together across all voters and the candidate with highest summed values is chosen to be the winner. Such a system is useful where the correct class does not appear at the top position of the classifier’s Softmax output. Ranking based systems are also useful in avoiding inconsistencies between continuous output values of different classifiers, since normalization of classifier outputs (scores, confidences, and posterior probabilities) is not trivial [29].

##### Max Rule

Given the class scores from all classifiers, where $g_{1},\;g_{2}..g_{R}$ are the classifiers and $C_{1},\;C_{2}...C_{m}$ are the classes, the Max rule assigns the test pattern to the class with the highest confidence score across all classifiers. Let $P_{i}^{j}\left(x\right)$ be the confidence score for class $C_{j}$ using classifier $g_{i}$ for a given feature pattern *x*. The final class is then decided as in Equation (7).

(7) $$\begin{array}{c} P_{l}^{\hat{g}}=\max P_{i}^{j}\left(x\right)\;\;\forall \;i,j \end{array}$$

thus, the feature pattern *x* is assigned the class *l* given by classifier $\hat{g}\in g_{i}\;\left(i=1,2..R\right)$.

In this work, the combination rules were applied on the uni-modular results of the separately trained dynamic signatures and EEG signals using the feature vectors $F_{S}$ and $F_{D}$, respectively.

#### 3.4.2. Person Verification

The verification of a queried person was performed by matching the identity against all reference templates that belong to the claimed identity. If the matching score was greater than some acceptance threshold th, the person was considered as genuine, otherwise they were rejected as a forger. The probability ($P_{i}$) of a test sequence (*X*) being a claimed identity (*i*) was matched with that user’s standard probabilistic threshold ($th_{i}$) as defined in Equation (8). If the condition in Equation (8) was satisfied, the person claim was considered as genuine.

(8) $$P_{i}\left(X\right)>th_{i}\left(X\right)\;where\;X\in \left\{EEG,Signature\right\}$$

In the multimodal scenario, this verification process was extended using the fusion rules described in Section 3.4.1 by fusing the outputs of the EEG and signature models. This combined score was matched against a threshold th for the genuine user to make a final decision.

## 4. Results

The performance of all systems was evaluated using five-fold cross validation, using 80% of the dataset for training and the remaining 20% for testing. Using this, we divided the dataset into five independent groups (or folds), held out one fold for testing, and performed training using the other four. The process was repeated five times with a different fold being used for testing each time. Finally, the average results of all folds are reported. Person identification results were evaluated for the 33 genuine users. Within each fold, 80% (eight signature and EEG samples) was kept for training and the remaining 20% (two signature and EEG samples) for testing. The same strategy was adopted for verification results, where the system was trained with 80% data of genuine users and tested using the remaining 20% plus forged data. In the verification phase, the decision of a genuine or forged user was governed based on a threshold value determined to maximize the true positive rate and minimize the false acceptance rate.

**Figure 6:**
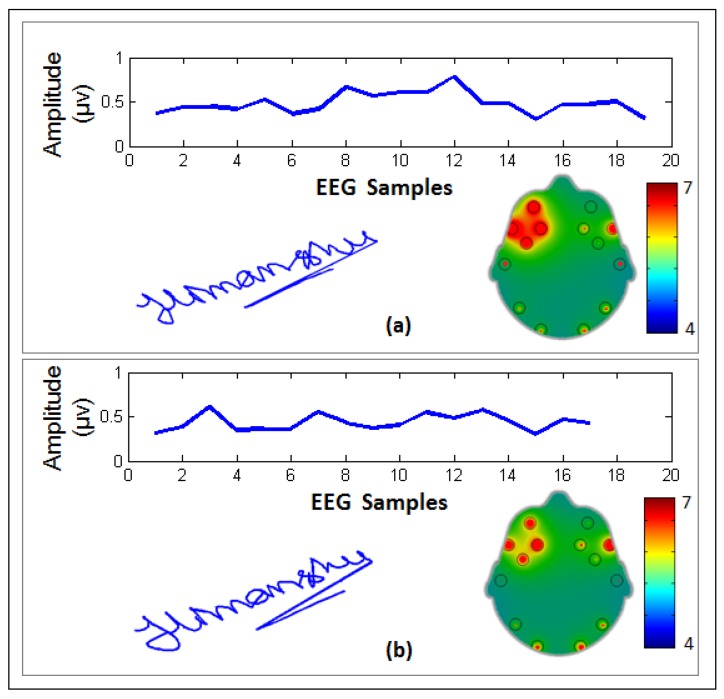
Examples of data samples from two different user: (**a**) online signature samples and (**b**) brain activity map EEG signals. Note that electrodes with larger red areas denote higher activity levels at those sites. Each EEG sample denotes ~7.8 ms.

**Figure 7:**
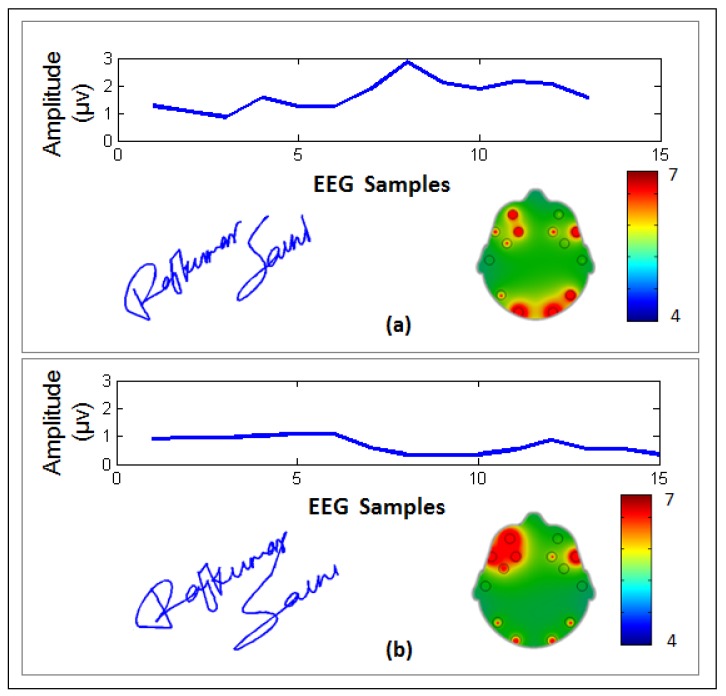
Example of the EEG signals, dynamic signature, and EMOTIV brain activity map associated with a (**a**) genuine user and (**b**) forgery attempt by another user. Note that electrodes with larger red areas denote higher activity levels at those sights.

An example of the EEG signals, signature samples, and brain activity (BA) map of a representative genuine user are shown in Figure 6 for two different signature samples. It can be seen that the BA map for this user shows high activity levels in the frontal lobe of the brain. For comparison, Figure 7 shows a comparison of a genuine user (Figure 7a) and a forgery attempt (Figure 7b). It can be seen that although the signature is quite good, the user was unable to imitate the EEG signals.

### 4.1. Person Identification Results

The person identification results were computed in two phases. First, the results were found using individual (unimodal) traits, i.e., dynamic signatures or EEG signals. Second, the identification results were computed using the decision fusion based (multimodal) approaches discussed in Section 3.4.1.

#### 4.1.1. Identification Using Dynamic Signatures

**Figure 8:**
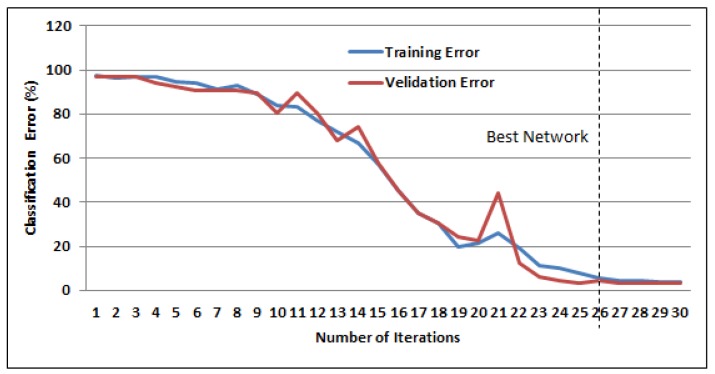
Bi-directional long short-term memory (BLSTM) training learning curve for dynamic signature trajectories showing error variation in training and validation data.

The decay in the training and validation errors of the unimodal signature-based BLSTM-NN are shown by the learning curve depicted in Figure 8. The decay in the validation errors slowed after 26 training epochs, thus training was stopped at this point. An average accuracy of 96.36% was recorded when the network was tested using dynamic signatures alone.

#### 4.1.2. Person Identification Using EEG Signals

**Figure 9:**
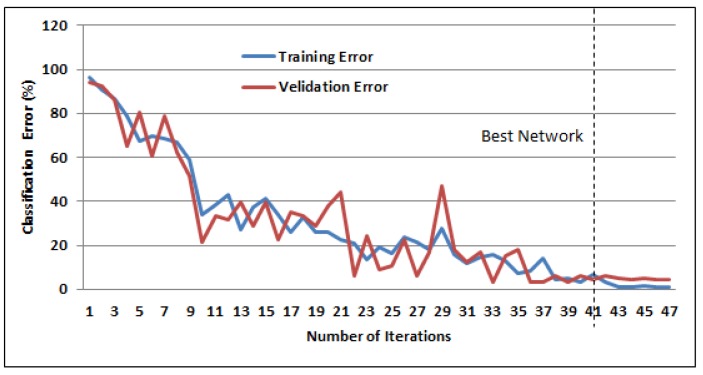
BLSTM training learning curve for EEG signals with training and validation data.

The variation in the training and validation error of the unimodal EEG-based BLSTM-NN during training are shown in Figure 9. Training was stopped when the variation in the validation error became roughly constant after 41 training epochs. A recognition rate of 97.57% was obtained when using the gamma features ($F_{D}$) comprised of all EEG sensors.

The identification accuracy was also computed for different subsets of brain lobes, namely, Left−Frontal, Right−Frontal, Full−Frontal, Temporal, Occipital and Parietal. A description of these lobes, the corresponding electrodes, and the resulting recognition rates are shown in Table 1, where the best single sensor accuracy of 73.36% was recorded from the Full−Frontal lobe of the brain.

**Figure 10:**
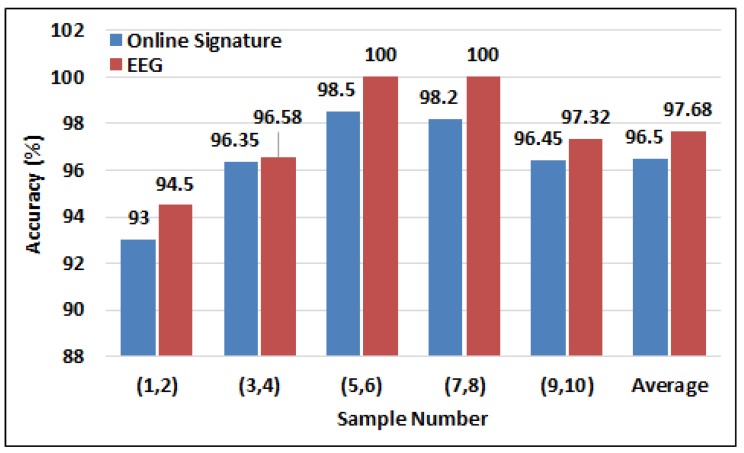
Analysis of person identification performance using dynamic signatures and EEG signals of the sequence of samples collected over time.

The average performance of the unimodal identification systems across the 10 samples collected from each participant is shown in Figure 10. It can be seen that the early and late sets of samples obtained lower accuracies than those in the middle. This may be in part due to learning or changes in concentration over time, or subjects may have become fatigued or disengaged after performing many signatures.

#### 4.1.3. Person Identification Using Multimodal Decision Fusion

**Figure 11:**
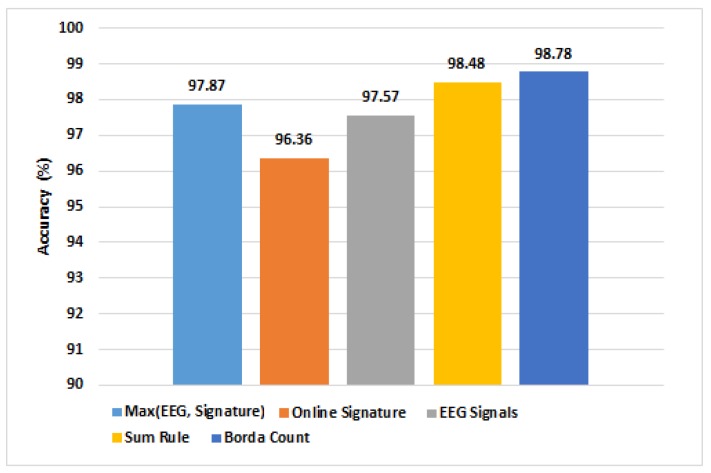
Person identification performance of the unimodal and decision fusion approaches.

The preceding sections have presented the unimodal results alone, but these systems were also combined, as discussed previously. A comparison of the identification performances of the unimodal and decision fusion approaches is therefore shown in Figure 11. Of particular note, an accuracy of 98.78% was obtained for person identification using the Borda count fusion technique to combine both signature and EEG.

### 4.2. Person Verification Results

**Figure 12:**
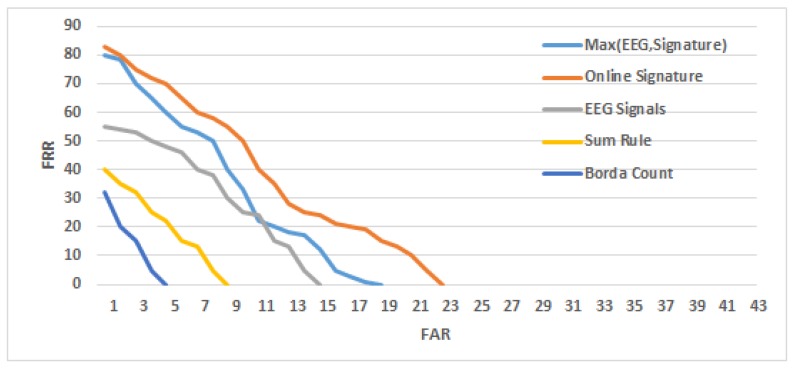
Detection error tradeoff curves for person verification using dynamic signature, EEG signals, and decision fusion approach.

In contrast to identification, the verification process evaluates a system’s ability to catch a would-be forgery. The verification rate of attempted imposters was calculated and reported using a detection error tradeoff (DET) curve, which relates the false acceptance (FAR) and false rejection rates (FRR), as shown in Figure 12. It can bee seen that the best results were achieved using the Borda count decision fusion verification model, with FAR of 3.75%. Comparatively, FARs of 14.91% and 22.5% were recorded when using the EEG and dynamic signature based verification models alone, respectively.

Verification results were also computed using the half total error rate (HTER) based security metric, which is defined as the average of FAR and false rejection rate (FRR). Here, HTER values were calculated at FRR equals to zero. HTERs of 1.87%, 11.25%, and 7.45% were recorded using the decision fusion, dynamic signature alone, and EEG-alone based security models, respectively.

Additional offline analyses were also conducted to evaluate the proposed approach on other publicly available datasets of EEG and signatures: Physionet [30] and MCTY DB1 [31], respectively. Physionet is a 64-channel EEG dataset of 109 users recorded for 60 s with two baseline tasks. The Physionet dataset was used by Fraschini et al. [32] to build a person identification system. The authors applied a band-pass filter and estimated the functional connectivity using the phase lag index (PLI) and the similarity was performed based on Euclidean distance. The methodology was applied to baseline tasks of eyes open (EO) and eyes closed (EC) yielding accuracies of 96.9% and 92.6%, respectively. For comparison, we applied the proposed BLSTM classification scheme to the same dataset and obtained person identification rates of 97.41% and 93.12% for the EO and EC cases, respectively. Similarly, we performed experiments on MCYT DB1 which is a dynamic signature database of 100 individuals, each performing 25 genuine and 25 forgeries. Recently, Riesen et al. [33] proposed a modified string edit distance matching algorithm based on a cost model, obtaining equal error rate (EER)s of 1.65% and 4.20% on random and skilled forgeries on MCYT database, respectively. Raul et al. [11] proposed the inclusion of number of strokes and signing time as important features to restrict forgery attempts yielding EERs of 0.54% and 3.6% on random and skilled forgeries, respectively. Because of the current protocol, no direct comparison could be made with the random forgeries case. Nevertheless, the proposed algorithm generalized well, obtaining a comparable EER of 4.01% for the skilled forgeries case.

## 5. Discussion

Over the years, the need for robust biometric systems to help stop unauthorized access to locations or digital information has increased substantially. Systems based on various biometric modalities (e.g., fingertips, signature, iris, etc.) have been proposed and employed [34]. However, many of the more convenient modalities are also prone to different security attacks such as artificial fingerprints, skilled forgers, etc. In general, signatures are the most widely acceptable means for person identification by many organizations and institutions, whether they be via conventional pen-and-paper or digital signatures on mobiles or tablets. Signed documents, regardless of form, can easily be forged by skilled forgers who can effectively and quickly imitate a genuine signature.

In this paper, we provide additional strength to signature based identification and verification systems by integrating the user’s brain activity (EEG) while performing their signature. EEG signals are considered as a robust biometric trait and have been successfully used in high security environments. With the emergence of mobile devices, EEG signals can now be captured at a distance through wireless connectivity and can be integrated into a variety of BCI applications for healthcare, remote monitoring, smart homes, etc. Here, we integrate EEG signals with dynamic digital signature because the signature strokes stimulate the EEG signals in the brain and provide a unique pattern of signals while signing, as depicted in Figure 2. Merging this temporal and attentional relationship between signature and EEG data results in a robust biometric system that is extremely difficult to forge.

**Figure 13:**
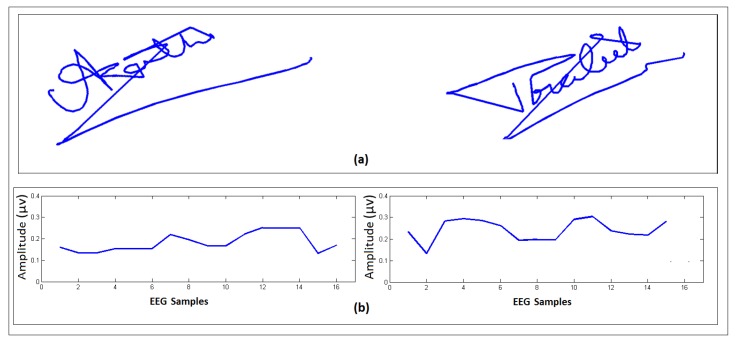
Error analysis: (**a**) Wrongly classified dynamic signature samples of two different users due to similar writing pattern; (**b**) wrongly classified EEG signals sample due to noisy data caused by unusual body movements.

Based on the unimodal algorithms, in total, nine dynamic signatures and eight EEG samples were wrongly classified during the identification process. For the signatures, very similar writing styles between two participants with the same number of characters in their signatures may have created confusion. An example of this scenario is depicted in Figure 13a. For the EEG signals, the samples may have been wrongly identified either due to a lack of concentration on the task, or due to extraneous head movements causing motion artefacts in the recorded EEG signals. These are nevertheless conditions that must be anticipated by such a system. It should be noted that, although these errors occurred in the unimodal case, most often, the other modality was correct. This motivates the further exploration of multimodal approaches, and the possibly incorporation of additional sensors, such as IMUs for movement detection.

Mobile phone are now ubiquitous across the globe and wearable technologies are showing promise that they will follow the same trends. As such, emerging approaches such as those proposed here may one day become feasible for a vast range of security applications, extending to smart-homes, health information, and scientific and military applications. With future research, the identification and verification accuracies could be further improved by employing robust multi-classifier combination methods and other neural network topologies. Other works should focus on exploring the stability of these signals during stressful and dynamic situations, and their effects on the robustness of both identification and verification.

## 6. Conclusions

In this paper, a mobile phone-based person identification and verification framework using dynamic signatures and EEG signals has been proposed. The EEG signals of 58 users were collected while they signed on a mobile phone screen. The identification and verification performance was computed using both unimodal schemes individually, and as a fused multimodal system comprised of a BLSTM classifier. An accuracy of 98.78% was achieved in person identification and a FAR of 3.75% was obained in person verification (for a true positive rate (TPR) of 100%) using the decision fusion-based classification scheme, outperforming previously reported architectures. 

## Figures and Tables

**Table 1:** Person identification performance on different brain lobes.

Brain Lobes	Electrodes Description	Accuracy (%)
Left-Frontal	AF3, F3, F7, FC5	69.21
Right-Frontal	AF4, F4, F8, FC6	73.36
Full-Frontal	AF3, AF4, F3, F4, F7, F8, FC5, FC6	73.36
Temporal	T7, T8	66.15
Parietal	P7, P8	66.15
Occipital	O1, O2	66.15
